# The Effects of Strength Training Tailored to Personalized Force-Velocity Curves on Speed and Change-of-Direction Ability of University Badminton Players

**DOI:** 10.5114/jhk/207300

**Published:** 2025-11-20

**Authors:** Yuer Shi, Wuwen Peng, Wenhao Qu, Yuanfu Luo, Duanying Li, Wenzhong Xue, Tao Chen, Jian Sun

**Affiliations:** 1 School of Athletic Training, Guangzhou Sport University, Guangzhou, Guangdong, China.; 2 Xinhe School, Baiyun District, Guangzhou, Guangdong, China.; 3 School of Wushu, Guangzhou Sport University, Guangzhou, Guangdong, China.; 4 Badminton Technical and Tactical Analysis and Diagnostic Laboratory, National Academy of Badminton, Guangzhou Sport University, Guangzhou, Guangdong, China.

**Keywords:** racquet sports, velocity-based, percentage-based, speed, resistance exercise

## Abstract

The purpose of this study was to examine the effects of velocity-based resistance training (VBRT) and percentage-based resistance training (PBRT) on speed and change-of-direction ability in university badminton players. Thirty-three university-level players were divided into VBRT, PBRT, and control (CON) groups, training twice weekly for eight weeks. The VBRT group adjusted loads based on individual load-velocity profiles (LVPs). Post-intervention, both VBRT and PBRT groups showed significant improvements in the10-m sprint test, T-test for agility, hexagon jump test, and shuttle change of direction test performance (all p < 0.05), with no changes in the CON group. VBRT led to greater performance gains, with no significant difference in perceived exertion between VBRT and PBRT despite higher absolute loads for VBRT.

## Introduction

Badminton is a net-based competitive sport dominated by skill, characterized by the core attributes of speed, accuracy, power, and agility (Zhang, 2019). In competition, the speed of a badminton shuttlecock can exceed 400 km/h, which places exceptionally high demands on an athlete's reaction speed and agility (Ramasamy et al., 2024). Additionally, athletes need to move quickly and agilely on the court to respond to shuttlecocks coming from any direction and perform actions such as sudden stops, rapid starts, and multidirectional changes of direction (CODs) during the shuttlecock-striking process (Li and Ding, 2021). Therefore, short-distance sprinting and CODs not only play a crucial role in the transition of the game tempo, but also act as decisive factors during critical moments of the match (Barrera-Domínguez et al., 2024).

As the demand for speed and agility continues to increase among badminton players, finding effective training methods to optimize these abilities becomes crucial. Traditional resistance training often utilizes percentage-based resistance training (PBRT), which determines training loads based on percentages of the athlete's one-repetition maximum (1RM). PBRT significantly enhances muscular strength and power, optimizes force output, improves movement efficiency and stability, and ultimately enhances sprint speed and COD ability (Suchomel et al., 2016). However, the 1RM test is time-consuming and complex to administer, and due to various factors such as training fatigue, sleep quality, life stress, and nutritional status, the 1RM value may fluctuate, making it difficult to accurately match the training load with the athlete's actual training needs(Baena-Marín et al., 2022). To address these issues, velocity-based resistance training (VBRT), as a modern training method, has garnered widespread attention. It dynamically adjusts training intensity and repetitions through real-time monitoring of load velocity, offering a more precise and individualized approach to training(Pagaduan and Pojskic, 2020).

VBRT *adjusts* training intensity based on the athlete's load-velocity profile (LVP) and uses the mean concentric velocity (MCV) of the first repetition as a performance metric to tailor training loads (Nevin, 2019). Precisely, by recording the changes in MCV under each load, the training load is adjusted accordingly, allowing for precise control of training intensity (González-Badillo et al., 2011). This approach ensures the individualization and precision of training loads, enabling athletes to perform movements at an optimal velocity, thereby effectively reducing energy expenditure and mitigating fatigue accumulation. Furthermore, VBRT dynamically optimizes training loads through real-time monitoring of movement velocity, enhancing the neuromuscular system's operational efficiency and better addressing the specific demands of speed and change-of-direction training for badminton athletes (Guerriero et al., 2018).

Existing research has shown that utilizing the individualized LVP for strength training regulation can effectively improve athletic performance in sports such as rugby, basketball, and handball (Orange et al., 2019; Zhang et al., 2024). Compared to the standard LVP constructed from extensive sample data, it offers a more prominent advantage in terms of personalization (Dorrell et al., 2020). Previous studies have demonstrated that the standard LVP can enhance performance in elite badminton players (Huang et al., 2023). However, there are currently no studies applying individualized LVPs to university badminton players to explore the impact of VBRT on speed and COD agility in this population. Therefore, this study applied VBRT using the force-velocity curve to compare the effects of VBRT and PBRT on speed and COD agility in university badminton players. It was hypothesized that eight weeks of VBRT training based on individualized load-velocity profiles would enhance the speed and agility of collegiate badminton players more effectively than PBRT.

## Methods

### Research Design

This study used a randomized parallel controlled experiment to assess the effects of eight-week, twice-per-week, individualized force-velocity curve-based VBRT on the shuttlecock speed and agility of university badminton players. Participants were randomly assigned to three groups via a Statistical Package for the Social Sciences (SPSS) generated sequence. All participants completed one familiarization session, two baseline testing sessions, and eight-week training. Testing sessions for all groups occurred at the same time to control for biological rhythms.

**Figure 1:**
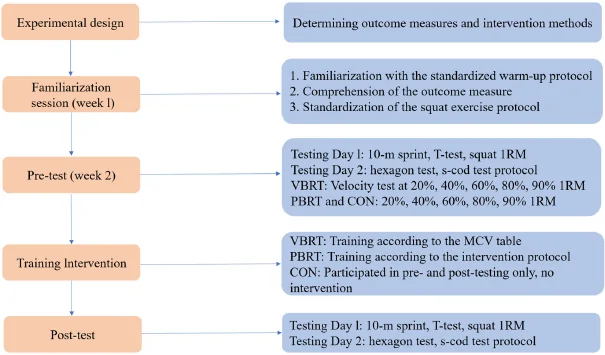
Experimental flow chart.

The familiarization session, held one week before baseline testing, introduced participants to the procedures, intervention protocols, testing indices, and equipment. Baseline speed and agility tests followed. The VBRT group then received load adjustments based on individual MCV, the PBRT group trained with a fixed cycle, and the control group (CON) had no intervention. Post-intervention speed and agility tests were conducted. Detailed information is provided in Figure 1.

### Participants

The participants were 33 third-year physical education students specializing in badminton. The sample size was determined using G*Power software (α = 0.05, power = 0.95, ES = 0.6), requiring a minimum of 27 participants. Considering the potential attrition, 33 participants were recruited. Inclusion criteria were as follow: (1) back squat 1RM ≥1.5 body weight; (2) no injuries in the past six months; (3) no conditions restricting physical activity; (4) male, aged ≥18; (5) at least two years of badminton training. Three participants withdrew due to illness or unrelated injury, leaving 30 for analysis (VBRT = 10, PBRT = 9, CON = 11). All participants provided informed consent. Detailed information is provided in Table 1.

**Table 1:** Basic information of the study participants.

Index	VBRT (n = 10)	PBRT (n = 9)	CON (n = 11)	F	*p*
Age (year)	21.9 ± 0.88	21.11 ± 0.78	21.45 ± 1.36	1.32	0.284
Body height (cm)	173.9 ± 6.01	177.11 ± 5.44	176.18 ± 4.96	0.884	0.452
Body mass (kg)	68.74 ± 6.25	67.16 ± 7.19	69.62 ± 5.66	0.378	0.689
Relative force	1.96 ± 0.13	1.95 ± 0.19	1.97 ± 0.16	0.047	0.954
Back squat 1RM (kg)	134 ± 9.66	130 ± 10.00	136.36 ± 4.52	1.479	0.246

Note: Values are reported as mean ± standard deviation; the differences among the three groups were obtained by one-way analysis of variance; p < 0.01 had a very significant difference, p < 0.05 had a significant difference, and p > 0.05 had no significant difference; VBRT: velocity-based resistance training; PBRT: percentage-based strength training group; CON: control group

This study was approved by the Ethics Committee for Human Experiments at the Guangzhou Sports University, Guangdong, China (approval No. 2023LLLL-78; approval date: 27 December 2023) and registered with the Chinese Clinical Trial Registry (ChiCTR2400087 593).

### Training Intervention

#### Intervention Approaches

The purpose of this experiment was to investigate the effects of two training methods, VBRT and PBRT, on the lower limb performance of collegiate badminton players. Given the complexity of the lower limb musculature, which includes significant muscle groups such as the gluteus maximus and quadriceps, enhancing the synergistic function of these muscles is crucial for improving lower limb performance. The back squat, which mobilizes both the hip and knee joints as a compound movement, is an excellent choice for developing the strength of lower limb performance (Schoenfeld, 2010).

#### Load for the VBRT Group

**Figure 2:**
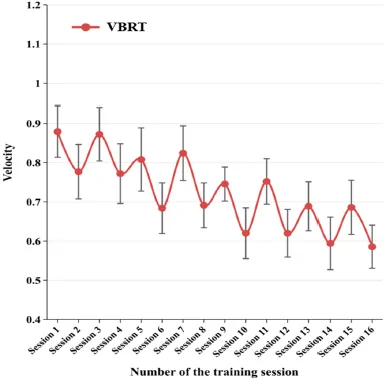
Graph motion velocity for VBRT.

After completing post-squat 1RM testing at 48 hours, the VBRT group was tested at 20%, 40%, 60%, 80%, and 90% 1RM, with MCV recorded for each set. Using the FORECAST function, a linear regression equation created the LVP, which was then converted into an MCV table for individualized training. For example, with a 1RM of 125 kg, velocities at 20%, 40%, 60%, 80%, and 90% 1RM were measured as 1.12, 0.86, 0.73, 0.50, and 0.38 m/s, respectively. The FORECAST function predicted velocities for intermediate loads to create the MCV profile (Figure 2).

Formal training used an undulating periodization model with daily load adjustments, alternating heavy and light loads weekly for better recovery and performance (MacDonald et al., 2012). Each session included four sets with 4-min rest intervals in between (Riscart-López et al., 2021). The VBRT group adjusted weights based on the individual LVP, aiming for an average set velocity within ±0.06 m/s; when this was exceeded, loads were modified by ±4–5% 1RM (Banyard et al., 2020). The PBRT group trained without load adjustments, while the CON group only completed pre- and post-tests (Table 2).

**Table 2:** Intervention protocols.

Session	VBRT Load Intensity	PBRT Load Intensity	Sets	Repetitions	Rest Interval
1	Speed Range Corresponding to 50% 1RM	50% 1RM	4	15	4 min
2	Speed Range Corresponding to 60% 1RM	60%1RM	4	15	4 min
3	Speed Range Corresponding to 50% 1RM	50%1RM	4	15	4 min
4	Speed Range Corresponding to 60% 1RM	60%1RM	4	15	4 min
5	Speed Range Corresponding to 55% 1RM	55%1RM	4	12	4 min
6	Speed Range Corresponding to 70% 1RM	70%1RM	4	10	4 min
7	Speed Range Corresponding to 55% 1RM	55%1RM	4	12	4 min
8	Speed Range Corresponding to 70% 1RM	70%1RM	4	10	4 min
9	Speed Range Corresponding to 65% 1RM	65%1RM	4	10	4 min
10	Speed Range Corresponding to 80% 1RM	80%1RM	4	6	4 min
11	Speed Range Corresponding to 65% 1RM	65%1RM	4	10	4 min
12	Speed Range Corresponding to 80% 1RM	80%1RM	4	6	4 min
13	Speed Range Corresponding to 75% 1RM	75%1RM	4	4	4 min
14	Speed Range Corresponding to 85% 1RM	85%1RM	4	4	4 min
15	Speed Range Corresponding to 75% 1RM	75%1RM	4	4	4 min
16	Speed Range Corresponding to 85% 1RM	85%1RM	4	4	4 min

#### Load for the PBRT Group

In the third testing module, to reduce the influence of confounding variables, during LVP testing for the VBRT group, the PBRT and CON groups trained at the corresponding loads. During the intervention, the PBRT group followed the training program exactly as planned (Table 2) without any load adjustments throughout the training period.

### Intervention Protocols

The experiment employed a wave-like periodization training design with daily fluctuations in load arrangement. Specifically, large and small loads were alternated in the two training sessions each week to allow better recovery and thus enhance athletic performance (MacDonald et al., 2012). Each session consisted of four sets with four-minute rest intervals between sets (Riscart-López et al., 2021) (Table 2).

### Outcome Measures

#### 10-m Sprint

The 10-m sprint test was conducted using a smart speed electronic timing system (Timing Systems, Brower, USA), with timing gates set at the starting and the finish line. Participants stood at the starting line and self-initiated the sprint. Each participant completed two trials with 3–5 minutes of rest intervals between subsequent attempts. The best time (measured to the nearest 0.01 s) was recorded for further analysis.

#### T-Test Protocol

The participant began at point A, sprinted to point B, and touched the cone with their right hand. They then shuffled laterally to point C and touched the cone with their left hand before finally shuffling to point D and touching the cone with their right hand.

### Hexagon Test Protocol

The participant stood in the center of a hexagon marked on the ground. Upon receiving the signal, they hopped with both feet from the center to each of the six sides in a clockwise direction, returning to the center after each hop. This sequence was repeated three times while maintaining the same direction. The test was performed twice, and the best recorded time (measured to the nearest 0.01 s) was used as the final result.

### S-COD Test Protocol

The specialized change of direction test was conducted as follows: the participant started at the center of a badminton court. Upon receiving the start command, they used “split-step” footwork to touch four cones in the following sequence: Forehand front → Backhand front → Backhand back → Forehand back. After touching each cone, they returned to a 40-cm circular starting area before proceeding to the next cone. This sequence was completed twice, totaling eight touches. The time was recorded (to the nearest 0.01 s) from the start to the completion of the second lap.

### Statistical Analysis

Data analysis was conducted using SPSS 26.0 and JASP 0.18.3. The Shapiro-Wilk test was used to assess normality, the Levene's test was employed to check homogeneity of variance, and the Mauchly's test was conducted to verify sphericity. Data following a normal distribution were presented as mean ± standard deviation (SD), while non-normal data were reported as median ± interquartile range. When Shapiro-Wilk or Mauchly's tests yielded *p* < 0.05, the Scheirer-Ray Hare test and Greenhouse-Geisser correction were applied, respectively. Pre-test data showed normal distribution and no significant group differences, thus repeated measures ANOVA was used. Independent *t*-tests analyzed VBRT and PBRT differences in the load and the RPE, while paired *t*-tests assessed pre- and post-test changes within groups. Two-way repeated measures ANOVA tested interaction effects, with Bonferroni-adjusted pairwise comparisons for significant interactions. Significant *p*-values, partial eta-squared, and Cohen’s *d* (as effect size measures) were reported where relevant. The rate of change was calculated as (post-test score − pre-test score) / pre-test score, with alpha set at 0.05.

## Results

### Training Data

**Figure 3:**
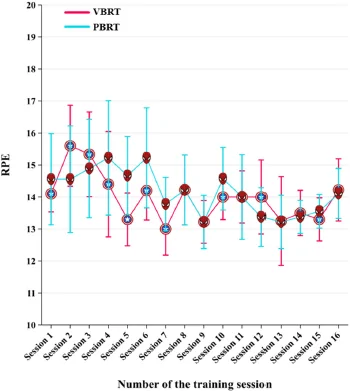
Graph of RPE change.

**Figure 4:**
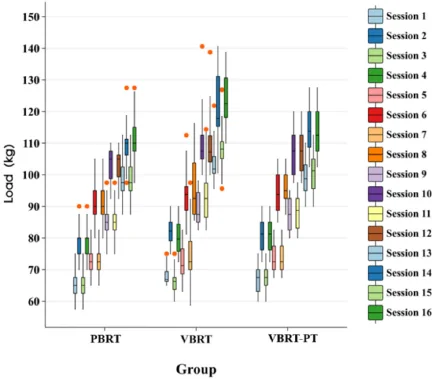
Graph of training load variation.

Figure 3 shows the average absolute load and RPE data for the two groups over 16 training sessions. The study found significant differences in the overall load between the VBRT and PBRT groups (*p* < 0.05) (Figure 4). In-depth analysis revealed significant load differences between the groups in the 15^th^ and 16^th^ sessions and in the 8^th^ week (*p* < 0.05). However, the overall RPE did not differ significantly between groups, with notable differences only in the 5^th^ session and the 3^rd^ week's RPE (*p* < 0.05) (Table 3)

**Table 3:** Characteristics of training loads.

Training Monitoring	Group	Session 1	Session 2	Session 3	Session 4
Load Intensity (kg)	VBRT	68.19 ± 3.29	82.19 ± 4.82	66.53 ± 4.71	79.94 ± 5.51
	PBRT	65 ± 5.00	79.17 ± 5.99	65 ± 5.00	79.17 ± 5.99
	VBRT-PT	67.25 ± 4.78	81.25 ± 5.8	67.78 ± 4.75	81.25 ± 5.80
Average Speed (m/s)	VBRT	0.88 ± 0.07	0.78 ± 0.07	0.88 ± 0.07	0.77 ± 0.08
	VBRT-PT	0.87 ± 0.08	0.77 ± 0.07	0.87 ± 0.08	0.77 ± 0.07
Number of Repetitions	VBRT	15	15	15	15
	PBRT				
RPE	VBRT	14.10 ± 0.57	15.60 ± 1.26	15.33 ± 1.32	14.40 ± 1.65
	PBRT	14.56 ± 1.42	14.56 ± 1.67	14.89 ± 1.54	15.22 ± 1.79
Load Intensity (kg)	VBRT	88.96 ± 5.74	111.25 ± 12.54	93.19 ± 9.44	111.25 ± 11.06
	PBRT	85.56 ± 6.47	102.86 ± 6.52	85.56 ± 6.47	103.13 ± 6.09
	VBRT-PT	87.50 ± 5.30	107.50 ± 7.91	88.50 ± 5.92	108.00 ± 7.62
Average Speed (m/s)	VBRT	0.75 ± 0.04	0.62 ± 0.06	0.75 ± 0.06	0.62 ± 0.06
	VBRT-PT	0.73 ± 0.08	0.6 ± 0.08	0.73 ± 0.08	0.6 ± 0.08
Number of Repetitions	VBRT	10	4	10	4
	PBRT				
RPE	VBRT	13.22 ± 0.67	14.00 ± 0.71	14.00 ± 0.82	14.00 ± 1.15
	PBRT	13.22 ± 0.83	14.57 ± 0.98	14.00 ± 1.32	13.38 ± 0.92
**Training Monitoring**	**Group**	**Session 5**	**Session 6**	**Session 7**	**Session 8**
Load Intensity (kg)	VBRT	72.50 ± 6.03	94.38 ± 8.67	74.56 ± 10.21	95.83 ± 10.15
	PBRT	72.50 ± 5.0	91.94 ± 6.59	72.50 ± 5.00	91.94 ± 6.59
	VBRT-PT	74.00 ± 5.03	94.75 ± 6.92	74.00 ± 5.00	95.83 ± 6.37
Average Speed (m/s)	VBRT	0.81 ± 0.08	0.68 ± 0.06	0.82 ± 0.07	0.69 ± 0.06
	VBRT-PT	0.82 ± 0.08	0.69 ± 0.08	0.82 ± 0.08	0.69 ± 0.08
Number of Repetitions	VBRT	12	10	12	10
	PBRT				
RPE	VBRT	13.30 ± 0.82	14.20 ± 0.92	13.00 ± 0.82	14.22 ± 1.09
	PBRT	14.67 ± 1.22	15.22 ± 1.56	13.78 ± 0.83	14.22 ± 1.09
Load Intensity (kg)	VBRT	104.53 ± 8.11	121.19 ± 12.23	107.94 ± 8.61	123.54 ± 10.01
	PBRT	98.06 ± 7.37	111.25 ± 8.45	98.06 ± 7.37	111.67 ± 8.00
	VBRT-PT	99.38 ± 6.37	115.00 ± 8.08	101.25 ± 7.10	115.00 ± 8.57
Average Speed (m/s)	VBRT	0.69 ± 0.06	0.6 ± 0.07	0.69 ± 0.07	0.59 ± 0.05
	VBRT-PT	0.64 ± 0.08	0.56 ± 0.08	0.64 ± 0.08	0.56 ± 0.08
Number of Repetitions	VBRT	4	4	4	4
	PBRT				
RPE	VBRT	13.25 ± 1.39	13.50 ± 0.71	13.30 ± 0.67	14.22 ± 0.97
	PBRT	13.22 ± 0.83	13.38 ± 0.52	13.56 ± 0.53	14.11 ± 0.78

Note: VBRT: velocity-based resistance training; PBRT: percentage-based strength training group; VBRT-PT: unadjusted data based on velocity-based resistance training; RPE: rating of perceived exertion

### Pretest Data

All test indicators showed no significant within-group differences pre-experiment (*p* > 0.05); thus, repeated-measures ANOVA was used for between-group comparisons.

### Intra-Group Comparison

After eight weeks, the VBRT group showed significant improvements in the 10-m sprint, T-test, and S-COD scores (*p* < 0.05). The PBRT group improved significantly in the hexagon test (*p* < 0.05). The CON group showed no significant changes in any test indicators (Table 4).

**Table 4:** Pre- and post-intervention indicators.

Index	Group	Pre	Post	Rate of Change (%)	Main Effect		$\eta_{p^{2}}$
					Group Effect	Time Effect	Group ×Time
Hexagon Test (s)	VBRT	13.02 ± 0.86	11.00 ± 0.75***	15.56	0.793ns	56.779***	0.455
	PBRT	12.44 ± 1.15	11.47 ± 0.50**	7.80			
	CON	12.56 ± 1.02	12.20 ± 1.09ns	2.85			
10-m sprint (s)	VBRT	1.96 ± 0.07	1.79 ± 0.07***	8.40	0.44ns	18.289***	0.463
	PBRT	1.95 ± 0.13	1.84 ± 0.06**	5.90			
	CON	1.89 ± 0.10	1.93 ± 0.11ns	2.16			
T-test (s)	VBRT	10.60 ± 0.47	9.98 ± 0.50***	5.93	3.868ns	20.144**	0.381
	PBRT	10.57 ± 0.35	10.30 ± 0.36*	2.60			
	CON	10.74 ± 0.41	10.75 ± 0.43ns	−0.13			
S-COD (s)	VBRT	20.75 ± 1.22	18.62 ± 1.03***	10.67	3.33ns	63.730***	0.450
	PBRT	21.09 ± 1.14	19.70 ± 0.90***	6.60			
	CON	20.84 ± 0.91	20.50 ± 0.76ns	1.63			

Note: */**/***/ns: indicate the comparison between before and after the intervention, where * indicates a significant difference, p < 0.05; ** indicates a very significant difference, p < 0.01; *** indicates significant difference with p< 0.001; ns indicates no significant difference with p greater than 0.05

### Intergroup Comparison

The results of the between-group comparisons showed no significant differences among the three groups in their 10-m sprint and S-COD performance. However, there were significant differences in their T-test and hexagon test scores. The post-hoc comparison results for the T-test for agility indicated the following Cohen's *d* effect sizes: VBRT > PBRT (*d* = −0.342), VBRT > CON (*d* = −1.074), and PBRT > CON (*d* = −0.731). Taken together, the ranking was summarized as VBRT > PBRT > CON. For the hexagon test scores, the Cohen's *d* effect sizes were: PBRT > VBRT (*d* = 0.057), VBRT > CON (*d* = −0.4), and PBRT > CON (*d* = −0.457). The overall ranking was expressed as PBRT > VBRT > CON. In summary, the results indicated that the VBRT group outperformed the PBRT and CON groups in the T-test for agility. In contrast, the PBRT group demonstrated superior performance compared to the VBRT and CON groups in the hexagon test (Table 5).

**Table 5:** Inter-group comparison among VBRT, PBRT, and CON groups.

Test index	Group		$\eta_{p^{2}}$	Cohen's *d*
10-m sprint	VBRT	PBRT	0.032	−0.201
		CON		−0.348
	PBRT	CON		−0.147
T-test	VBRT	PBRT	0.223	−0.342
		CON		−1.074
	PBRT	CON		−0.731
Hexagon test	VBRT	PBRT	0.055	0.057
		CON		−0.400
	PBRT	CON		−0.457
S-COD	VBRT	PBRT	0.198	−0.716
		CON		−0.990
	PBRT	CON		−0.274

Note: VBRT: velocity-based resistance training; PBRT: percentage-based strength training group; CON: control group

## Discussion

### Sprinting Ability

Short-distance sprint speed is a critical athletic performance ability for badminton players (Cabello and González-Badillo, 2003). Compared to PBRT, VBRT resulted in significantly greater improvements in sprint speed among university badminton players, which is consistent with previous esearch (Baena-Marín et al., 2022).The advantage of VBRT may stem from its personalized load adjustments, allowing athletes to maintain a high and consistent repetition speed throughout training, ensuring that the mean velocity within the group fluctuates no more than 0.06 m/s within the set LVP load range. This precise load control ensures that the training load is highly aligned with the athlete's actual capacity, thereby maximizing motor unit recruitment, especially the activation of high-threshold motor units and fast-twitch muscle fibers (Baena-Marín et al., 2022). Additionally, Lahti et al. (2020) noted that VBRT, through high-intensity training, induced a shift in the muscle fiber type towards IIa fast-twitch fibers, which is crucial for enhancing explosive power and sprinting ability (Lahti et al., 2020).

In contrast, VBRT adjusts the load intensity for each set based not only on the individual's LVP and speed loss but also by flexibly modifying training loads, sets, and repetitions to match the athlete's real-time performance (de Hoyo et al., 2021). This approach enhances the personalization of and adaptability to training, maximizing training effectiveness while reducing the risk of overtraining and injury (Greig et al., 2020). Through real-time load adjustments, VBRT effectively prevents the accumulation of fatigue due to excessive loads, allowing athletes to maintain high movement efficiency, optimize training stimuli, enhance central nervous system activation, and promote more efficient muscle Adenosine Triphosphate (ATP) synthesis, ultimately leading to significant improvements in short-distance sprinting ability (Shi et al., 2022).

However, the PBRT group failed to effectively account for changes in the athletes' physiological states during long-term training cycles. Previous research has shown that, as strength increases and fatigue accumulates over time, baseline 1RM measurements often fail to reflect an athlete's true maximum strength accurately. This results in a mismatch between the prescribed load and the athlete's actual capabilities. Therefore, PBRT does not effectively accommodate changes in the athlete's condition, lacks real-time load adjustments, and fails to adequately stimulate the adaptability of the nervous system, limiting the athlete's potential in short sprints (González-Badillo and Sánchez-Medina, 2010). This fixed-load training model does not achieve optimal training effects in high-intensity, dynamic movements such as sprints, mainly when there are significant fluctuations in the athlete's fatigue state, leading to an imprecise match between training loads and capability, which hampers improvements in explosive power and sprinting ability.

Therefore, compared to PBRT, VBRT, through dynamic load adjustments, is better able to match the athlete's actual capabilities, thereby enhancing sprint speed and agility. This adaptive mechanism allows VBRT to demonstrate greater effectiveness in optimizing athletic performance, particularly in sports that require rapid explosive power and precise changes of direction.

From a fatigue quantification perspective, Held and colleagues (2021) found through surveys that athletes exhibited greater recovery capacity and lower stress levels within 24 to 48 hours after engaging in VBRT compared to PBRT (Held et al., 2021). This increased recovery and reduced stress enable athletes to maintain action speeds and activate fast-twitch fibers more effectively during VBRT sessions. Although both VBRT and PBRT effectively enhance speed, their mechanisms of muscle activation differ: VBRT optimizes action patterns by monitoring movement speed in real-time and adjusting training loads accordingly, efficiently activating neurons and significantly enhancing neural response and coordination. This method not only increases the firing frequency and efficiency of neurons, but also promotes the coordinated action of multiple muscle groups, reducing unnecessary energy expenditure and ensuring the smoothness and accuracy of movements. In contrast, PBRT imposes more significant mechanical and metabolic stress on muscles by increasing the number of repetitions and sets, leading to micro-damage in muscle fibers and the accumulation of metabolites, which stimulates muscle repair and growth, thereby enhancing muscle volume and strength, and consequently improving speed (Liao et al., 2021).

### Change of Direction

Previous research has shown a significant correlation between COD ability and badminton match performance, with a correlation coefficient as high as 0.83, indicating that COD directly influences match outcomes for badminton players (Sekulic et al., 2013). This study's results revealed that VBRT significantly enhanced collegiate badminton players' performance in the T-test for agility, the hexagonal jump test, and ten low-center runs, with outcomes superior to those achieved with PBRT.

In this study, the change of direction tests employed included the T-test, the hexagonal jump test, and a sport-specific ten-trial low-center quadrangular run, which are primarily associated with sprint times. According to the research by Young et al. (2015), 57% of COD performance can be explained by sprint performance and muscle strength, highlighting the significant roles of these factors in influencing COD (Spiteri et al., 2014; Young et al., 2015). Research by Hernández-Davó et al. (2021) and Loturco et al. (2018) also indicates that directional changeability is primarily affected by sprinting capabilities. Baena-Marín et al. (2022) have demonstrated that, compared to PBRT, VBRT can more effectively enhance strength and speed, which may be the primary reasons for improvements in COD.

The results of the hexagonal jump test in this study are consistent with the findings of previous research (Zhang et al., 2023). They are closely linked to enhanced proprioception and improved neural adaptation. The hexagonal jump test requires athletes to perform rapid movements in multiple directions, effectively stimulating the proprioception of the lower limbs and gluteal muscles. This study utilized specific squat training protocols that directly enhanced the proprioceptive abilities of the knee joints, hips, and the trunk. These exercises contribute to athletes' enhanced perception of the body position and the movement state, thereby effectively improving their postural control capabilities (Zhang et al., 2023). Additionally, the hexagonal jump test necessitates that athletes quickly jump in and out of the hexagonal area, demanding rapid force application within short duration. VBRT enhances muscular power, enabling athletes to generate more force in a brief period of time, thus significantly improving performance in the hexagonal jump test (Tomljanović et al., 2011).

Secondly, neural adaptation and the enhanced recruitment of motor units may be mechanisms contributing to improved COD (Aagaard et al., 2002). VBRT maximizes the activation of motor units, particularly those of high-threshold and fast-twitch muscle fibers, enhancing the output of muscle speed and power. In contrast, the training velocity and neural activation intensity of PBRT may not match those of VBRT. Additionally, enhancing COD ability requires the development of rapid strength, increased eccentric strength in thigh muscles, and the ability of leg extensors to swiftly transition from eccentric to concentric muscle action (Miller et al., 2006). In tests of change-of-direction ability, isometric strength is crucial for optimizing triple extension (synchronous extension of the knee, hip, and ankle joints) because it helps maintain proper alignment of the lower limbs and enables quick acceleration after changing direction (Spiteri et al., 2015). Studies have found that COD ability correlates with eccentric strength of the knee flexors and maximum eccentric strength of the lower limbs, and the adaptability of eccentric training may have higher specificity to the speed of eccentric loads (Spiteri et al., 2014). During VBRT, high-threshold motor units are recruited selectively and synergistically in the eccentric phase, generating greater eccentric overloading, causing more substantial damage to muscle fibers, and triggering a stronger metabolic response.

This study has several limitations. Firstly, the relatively limited skill level of the participants may have contributed to significant improvements in athletic performance, which could, to some extent, affect the generalizability of the findings. Additionally, due to experimental constraints, the study only assessed the relationship between the external load and athletic performance without considering physiological and biochemical markers, thus limiting the observation of the internal load and physiological changes.

## Conclusions

For university badminton players, VBRT showed more significant improvements in the 10-m sprint, T-test, and S-COD performance, while PBRT showed better gains in the hexagon test performance. Additionally, both groups reported similar perceived exertion, though the VBRT group had a higher absolute training load.
